# Surveying the Homogeneity of a Molecular Electrocatalyst Embedded in a Metal‐Organic Framework Using Operando Characterization

**DOI:** 10.1002/cssc.202501380

**Published:** 2025-10-14

**Authors:** Marlene E. Hoefnagel, Jan S. D. Rodriguez, Sergi Campos‐Jara, Oleg Usoltsev, Dennis G. H. Hetterscheid, Sheena Louisia

**Affiliations:** ^1^ Leiden Institute of Chemistry Leiden University Einsteinweg 55 2333 CC Leiden The Netherlands; ^2^ CELLS‐ALBA Synchrotron Radiation Facility Cerdanyola del Vallès 08290 Barcelona Spain; ^3^ Laboratoire Interfaces et Systeèmes Electrochimiques (LISE) Sorbonne Université, CNRS 75005 Paris France

**Keywords:** homogeneity, metal‐organic frameworks, molecular electrochemistry, operando X‐ray absorption spectroscopy, oxygen reduction reaction

## Abstract

Homogeneous catalysis generally yields low catalytic current densities due to the small number of catalytic centers at the electrode surface. Incorporating molecular catalysts in metal‐organic frameworks (MOFs) has been proposed as a viable approach to immobilize them on electrodes, increasing current densities. In addition, molecular catalysts do not always remain in their homogeneous state, sometimes partially taking on a more heterogeneous character, which challenges the clear identification of the active species. Despite the risk of homogeneity loss, most studies on molecular catalysts embedded in MOFs have so far overlooked the possibility of heterogeneous deposit formation during electrocatalysis. In this work, a more comprehensive study on the changes of homogeneity exhibited by an MOF‐embedded molecular catalyst is presented. The Cu species formed in the NU1000|Cu‐tmpaCOOH MOF before, during, and after the oxygen reduction reaction using operando X‐ray absorption spectroscopy are investigated. The initial Cu^2+^ catalyst forms Cu^0^ clusters of diameter <2 nm upon application of a reductive potential. This work demonstrates that for Cu‐based molecular catalysts embedded in MOFs, it is essential to account for the possible changes in a molecular catalyst's homogeneity, regardless of the catalytic benefits its supporting structure might grant.

## Graphical abstract

A detailed study of the homogeneity changes exhibited by a molecular Cu catalyst embedded in a MOF during the oxygen reduction reaction. Operando X‐ray absorption spectroscopy reveals a transformation from a molecular Cu^2+^ catalyst to Cu^0^ clusters <2 nm, emphasizing the risk of attributing high catalytic performance to molecular catalysts that have lost their homogeneity, regardless of their hosting structure.

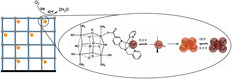

## 1 Introduction

Due to their uniform distribution in a solution, homogeneous catalysts allow for a more precise control over a given reaction compared to heterogeneous materials, allowing for the high selectivity and efficiency of a chemical reaction. Successful examples of homogeneous catalysis include Heck, Negishi, and Suzuki coupling reactions, olefin metathesis, and enantioselective hydrogenation reactions.^[^ ^1^, ^2^, ^3^, ^4^, ^5^ ^]^ Although it is relatively easy to gather mechanistic information, care should be taken that these homogeneous systems do not degrade to clusters or nanoparticles under catalytic conditions, leading to ambiguity regarding the true active species.^[^ ^6^, ^7^, ^8^ ^]^ These problems particularly arise when harsh reaction conditions are applied in, for example, an electrochemical environment. Water oxidation catalysts based on Fe, Ir, and Co were found to form FeOx, IrOx, and CoOx deposits, respectively.^[^ ^9^, ^10^, ^11^ ^]^ Furthermore, Cu‐based molecular catalysts are regularly found to form metallic Cu deposits, as shown in the case of the Cu(DAT) (DAT = 3,5‐diamino‐1,2,4‐triazole) catalyst for the oxygen reduction reaction (ORR).^[^ ^12^ ^]^ Similarly, a Cu–phthalocyanine catalyst for the CO_2_ reduction reaction (CO_2_RR) was found to form Cu clusters in situ, which was associated with the observed selectivity toward methane.^[^ ^13^ ^]^ Several techniques are being employed to study the homogeneity of a catalyst, that is, the extent to which it remains a molecular catalyst during the reaction.^[^ ^7^, ^14^, ^15^ ^]^ For example, it can be evaluated electrochemically by measuring the cyclic voltammetry (CV) of an electrode used for catalysis with a homogeneous catalyst in a clean electrolyte solution. If this CV exhibits redox features not belonging to the bare electrode itself, this activity originates from deposits on the electrode. Additionally, metal ion scavengers can be used to bind metal ions that have dissociated from their ligands. Furthermore, homogeneity can be characterized by spectroscopic techniques, such as X‐ray photoelectron spectroscopy,^[^ ^16^ ^]^ UV–vis absorption spectroscopy,^[^ ^17^ ^]^ mass spectrometry,^[^ ^18^ ^]^ nuclear magnetic resonance,^[^ ^19^ ^]^ and X‐ray absorption spectroscopy (XAS).^[^ ^20^ ^]^

In recent years, reports of homogeneous catalysts immobilized in metal‐organic frameworks (MOFs) as a strategy to improve their stability have become more frequent.^[^ ^21^, ^22^, ^23^, ^24^ ^]^ MOFs are porous 3D coordination polymers that consist of a highly symmetrical network of metal nodes and organic linkers. They are an interesting class of materials for electrocatalysis due to their high porosity and resulting high active surface area. Simultaneously, these materials may allow for more control over reactivity through confinement of active site, reactants, and products.^[^ ^8^, ^25^, ^26^, ^27^, ^28^ ^]^ The possibility to infinitely vary the structure of the framework by alterations of the linkers and nodes makes them interesting materials to study first and second coordination sphere effects on catalysis.^[^ ^29^, ^30^, ^31^, ^32^ ^]^ Additionally, incorporating homogeneous electrocatalysts into MOFs reduces the number of catalysts needed to obtain high catalytic currents and greatly facilitates catalyst recycling. Molecular catalysts can be incorporated into MOFs by various methods.^[^ ^33^ ^]^ The MOF linker itself can be a molecular catalyst,^[^ ^34^, ^35^ ^]^ the catalyst can be trapped inside the pore during MOF synthesis,^[^ ^36^ ^]^ or it can be bound to the linker or node by post‐synthetic modification methods.^[^ ^37^, ^38^ ^]^

Although immobilizing a homogeneous catalyst in an MOF presents, in theory, multiple advantages, few reports comment on the homogeneity of molecular catalysts immobilized in MOFs. However, this is of utmost importance given the tendency of many molecular catalysts to form heterogeneous deposits.^[^ ^39^ ^]^ The effects of high local concentrations of catalysts, reactants, and protons in a confined space are largely unexplored yet make these MOF‐embedded systems potentially vastly different from their homogeneous equivalents. This is especially important to consider for Cu‐based molecular catalysts because of the low energetic barriers and, therefore, fast kinetics in ligand exchange reactions.^[^ ^40^ ^]^ When Cu ions are pulled from the equilibrium by the accumulation of Cu or CuO_*x*_ particles, rapid decomposition of homogeneous Cu catalysts may occur. These equilibria may be highly dynamic and shift with the applied reaction conditions.^[^ ^26^ ^]^ Well‐performing molecular catalysts based on Cu have been developed for the CO_2_ reduction,^[^ ^41^, ^42^ ^]^ hydrogen evolution,^[^ ^43^, ^44^ ^]^ water oxidation,^[^ ^45^, ^46^ ^]^ and ORRs.^[^ ^47^, ^48^ ^]^ However, various reports have shown that the molecular Cu catalyst is not always the active species.^[^ ^12^, ^13^, ^49^ ^]^ In the works of Fontecave^[^ ^49^ ^]^ and Wang,^[^ ^13^ ^]^ Cu^0^ clusters were observed under operando conditions that were not visible with ex situ characterization. In both cases, a Cu^2+^ species was observed before and after the reaction and Cu^0^ species with distinguishable Cu–Cu distances during CO_2_ reduction. Fontecave and coworkers developed an N‐doped carbon material with single‐atom Cu catalysts for the CO_2_RR and suggested the metallic Cu clusters formed in situ are the true active species.^[^ ^49^, ^50^ ^]^ Similarly, Wang identified Cu clusters formed in situ from Cu–phthalocyanine as the active catalysts with a high selectivity to methane during the CO_2_RR. These cases highlight the dynamic nature of Cu‐based catalysts and thus the importance of operando characterization to monitor these processes. These dynamic effects are still expected when a molecular catalyst is incorporated in an MOF. Yet, to the best of our knowledge, no XAS characterization of MOF‐embedded molecular catalysts has been reported so far.

In a previous study, we incorporated a Cu‐tmpaCOOH catalyst for the ORR in the Zr‐based NU1000 MOF and thereby largely improved the reusability and catalytic stability of the MOF‐embedded catalyst compared to when dispersed in a homogeneous solution.^[^ ^51^ ^]^ NU1000 is selected as a very stable platform that itself is not redox active, allowing for evaluation of the performance of Cu‐tmpaCOOH in the absence of background reactivity. However, CV experiments after the catalytic reaction show the presence of a new redox couple. This suggests that there may be a different active species in the MOF. However, the presence of Cu‐tmpaCOOH was shown to be crucial to obtain any catalytic activity. Given the dynamic properties of Cu species, the in situ formation of Cu clusters observed by other groups, and our previous findings on the NU1000|Cu‐tmpaCOOH MOF, it is clear that operando techniques are required to 1) identify the active species present during the reaction and 2) understand the mechanisms by which a molecular Cu catalyst in MOFs operates. In this work, we evaluate for the first time the homogeneity of a molecular catalyst embedded in a MOF before, during, and after electrocatalysis using ex situ and operando XAS.

## 2 Results and Discussion

**Figure 1:**
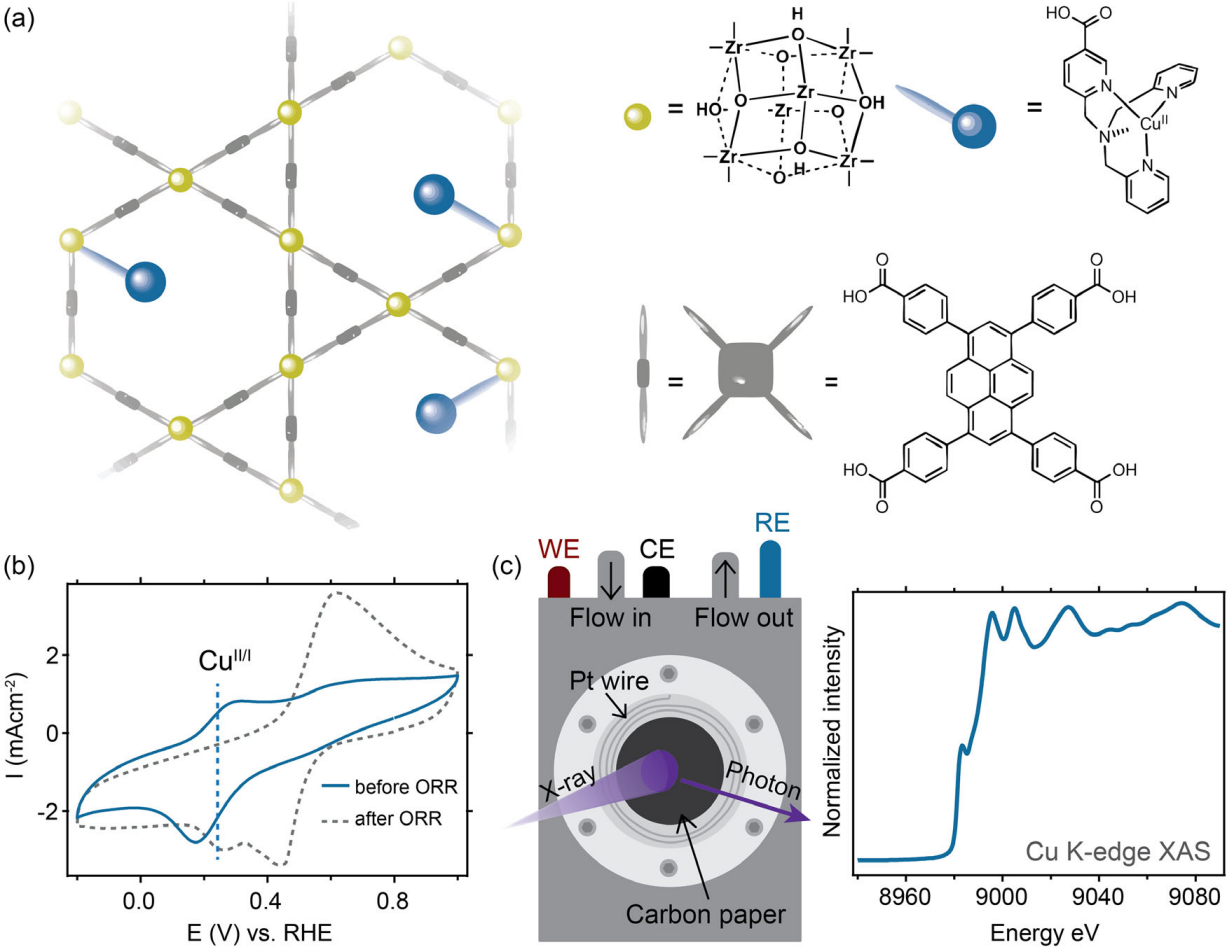
Spectroscopic investigation of NU1000|Cu‐tmpaCOOH. a) Schematic image of the NU1000|CutmpaCOOH structure and b) its CV at 50 mV s^−1^ under Ar before (blue) and after electrocatalysis at 0.3 V vs. RHE for 6 h (gray dash). c) Schematic representation of the operando XAS experiment.

Cu‐tmpaCOOH (tmpa = tris(2‐pyridylmethyl)amine) was synthesized and incorporated into the NU1000 MOF by solvent‐assisted ligand incorporation as described in our previous work to form the NU1000|Cu‐tmpaCOOH MOF (**Figure** **1**a).^[^ ^51^ ^]^ All measurements in the current work were performed using an MOF with a loading of 0.8 Cu centers per Zr node. We used the MOFs from the same batch that was previously described and characterized in ref. [47]. The CV of this MOF under Ar atmosphere shows a redox couple at 0.24 V vs. RHE (Figure 1b) that is assigned to the Cu^II/I^ redox couple. When exposed to an O_2_ atmosphere, the CV reveals a catalytic current with an onset potential of 0.33 V vs. RHE and a current density of −40 mA cm^−2^_geo_ (Figure S1a, Supporting Information). Chronoamperometry (CA) at 0.3 V vs. RHE (Figure S1b, Supporting Information) gives a stable current for at least five consecutive days.^[^ ^51^ ^]^ After electrolysis, a new irreversible redox couple is observed at 0.52 V vs. RHE in the CV under Ar (Figure 1b), indicating the formation of a new species. A very similar irreversible redox couple at 0.45 V vs. RHE was found by Hupp and coworkers in their work on Cu nanoparticles installed in NU1000.^[^ ^52^ ^]^ In their work, Cu(II) ions were installed solvothermically and nanoparticles formed by CA at −0.57 V vs. RHE for 20 min. Those nanoparticles were identified using transmission electron microscopy and reached a ≈5 nm diameter. The redox couple associated with these nanoparticles is similar to what we find after catalysis for NU1000|Cu‐tmpaCOOH. However, the mechanism behind the formation of these nanoparticles, which were identified ex situ, and their relevance to catalytic activity were not addressed. Therefore, operando XAS was applied in this work to uncover what species are formed during CA in our NU1000|Cu‐tmpaCOOH MOF. Unlike microscopic methods, operando XAS can 1) estimate whether very small (<5 nm) Cu particles are indeed formed under electrocatalytic conditions, 2) identify the nature of their potential‐dependent character, and 3) determine what structure corresponds to the redox couple formed only after electrocatalysis.

**Figure 2:**
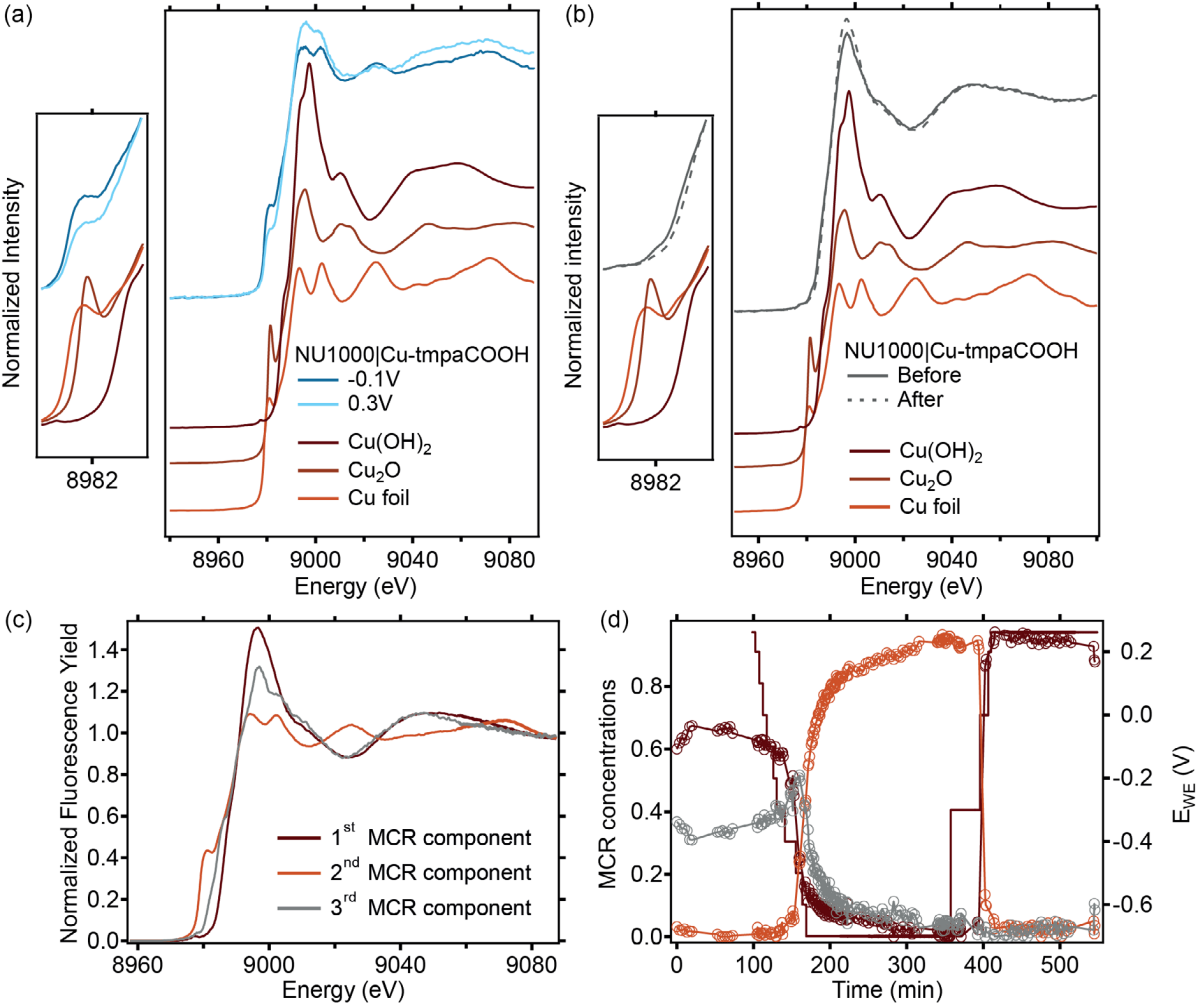
Cu K‐edge XANES of NU1000|Cu‐tmpaCOOH. a) Operando XAS measurements during CA under O_2_ atmosphere at 0.3 V vs. RHE (light blue) and −0.1 V vs. RHE (dark blue). b) In situ measurements at OCP before (gray) and after (gray intermittent) CA. Insets present the zoom of the pre‐edge region. The offset spectra of Cu foil, Cu_2_O, and Cu(OH)_2_ standards (reds) are given for qualitative comparison. c) MCR fitting of the NU1000|Cu‐tmpaCOOH at −0.1 V vs. RHE under O_2_ atmosphere yields three distinct components. d) The relative ratio of the three MCR fitting components as a function of time and the applied potential indicates the progressive change in the distribution of Cu states in the sample. An irreversible loss of the third MCR component (gray) is observed after carrying out electrolysis at −0.1 V vs. RHE for ≈3 h.

The MOF was dispersed in an ink containing carbon black, Nafion, and acetone as previously reported.^[^ ^51^ ^]^ The as‐prepared ink was drop casted onto a carbon paper working electrode used for operando XAS measurements in fluorescence mode. Although the density of Cu was too low to be detected with scanning electron microscopy and elemental dispersion X‐ray spectroscopy (Figure S2, Supporting Information), the acquisition of the fluorescence intensity at 9420 eV (i.e., well above the Cu K‐edge main edge jump) allowed us to roughly determine the dispersion of Cu over the electrode (Figure S3, Supporting Information). An electrochemical cell (Figure 1c and S4, Supporting Information) with a Kapton window was used with a constant flow of O_2_‐ or He‐saturated 0.1 M phosphate buffer pH 7 (Figure S5, Supporting Information). X‐ray absorption near‐edge spectroscopy (XANES) of the as‐prepared NU1000|Cu‐tmpaCOOH yields two pre‐edge features at 8977 and 8982 eV, which qualitatively coincide with the pre‐edge features of Cu(OH)_2_ and Cu_2_O, respectively (**Figure** **2** and S6, Supporting Information). However, the linear combination fitting (LCF) of the sample using those two references does not yield a good fitting (Figure S7a, Supporting Information). The addition of Cu^0^ foil as an additional reference also does not help with the quality of the fit (Figure S7b, Supporting Information). Qualitatively, NU1000|Cu‐tmpaCOOH closely resembles the XANES spectrum of Cu(OH)_2_ with perhaps a small Cu^0^ contribution (Figure S7c, Supporting Information). This strongly implies a Cu^2+^‐dominant character of the Cu sites in the catalyst. Once placed inside the electrochemical XAS cell and exposed to the O_2_‐saturated electrolyte at open‐circuit potential (OCP), we do not observe any noticeable change in the XANES features (Figure S8, Supporting Information). This suggests that the chemical state of the Cu sites existing in the as‐prepared catalyst remains stable at OCP. A succession of XANES scans measured for 40 min does not indicate any time‐dependent change (Figure S9, Supporting Information). This suggests that beam‐induced damage is negligible if present at all. We applied a more negative potential once the absence of beam‐induced change was confirmed. As soon as 30 mV negative of the onset potential, at 0.33 V vs. RHE, we already observed the formation of Cu^0^ species (Figure 2a). Similar to the sample before electrolysis, LCF using Cu^0^ foil and Cu(OH)_2_ instead of Cu_2_O yields better fits. Interestingly, at 0.3 V vs. RHE, the LCF analysis already indicates a 49:51 Cu^0^:Cu^2+^ ratio (Figure S10, Supporting Information). Taken more negative to –0.1 V, an even greater Cu^0^ fraction of 78% is measured (Table S1, Supporting Information). After returning to OCP, we measure a post‐electrolysis spectrum nearly identical to the pre‐electrolysis one (Figure 2b). Similarly to before electrolysis, the Cu species in the MOF exhibit a Cu^2+^ dominating oxidation state, as evidenced by the pre‐edge still observed at 8977 eV. However, the pre‐edge feature previously observed at 8982 eV is no longer visible. This suggests that although the dominant Cu state is mostly unchanged, a structural rearrangement has occurred during the ORR. Multivariate curve resolution (MCR) analysis was applied to better understand the chemical transformation of our system and verify the observations teased out using LCF analysis (Figure 2c). According to the imperfect fitting observed with LCF and given the complexity of matching the coordination environment of the Cu complex, it is not surprising that LCF is insufficient to represent the mixture of phases changing as a function of the applied potential. We assume that three main components must define the state of the molecular catalyst in the MOF (Figure S11, Supporting Information). MCR analysis identifies two components that are indeed nearly identical to the XAS features of Cu^0^ in a Cu foil and of Cu^+2^ in Cu(OH)_2_ (Figure S12, Supporting Information). The third component exhibits a main edge situated between the Cu^+1^ and Cu^+2^ states. However, its absorption features are very dissimilar to any common standards. This unknown third component may be attributed to the MOF‐embedded Cu(II)‐tmpaCOOH molecular catalyst still partially coordinated to a triflate anion (OTf^−^) used in its synthesis, or to a H_2_O molecule. Monitored as a function of the applied potential and time, the oxidation state of the Cu molecular catalyst is progressively reduced at −0.1 V to a Cu^0^‐only phase after 2 h of CA (Figure 2d). When returned to OCP after the CA measurement, the third MCR component initially present (≈35%) is no longer observed. Only the Cu^+2^ phase present at the beginning (≈75%) is recovered at OCP and represents nearly the sole phase in the system. Such a change corroborates the hypothesis that the molecular catalyst undergoes an irreversible structural transformation after being subject to electrocatalytic reaction conditions.

**Figure 3:**
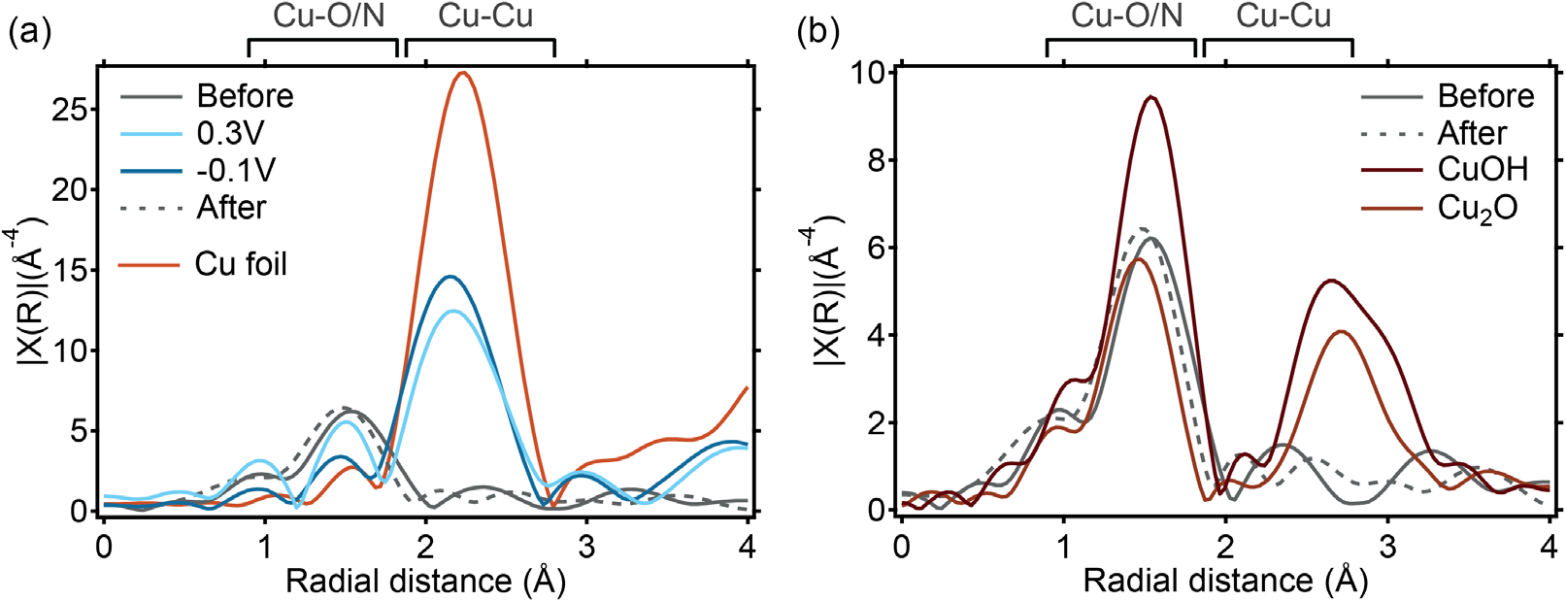
Cu K‐edge EXAFS of NU1000|Cu‐tmpaCOOH in R‐space. a) Phase‐uncorrected FT‐EXAFS spectra before (gray), after (gray intermittent), and during CA at 0.3 V vs. RHE (light blue) and −0.1 V vs. RHE (dark blue). Cu foil (red) is plotted to indicate the usual radial distance of Cu—Cu in bulk metallic Cu^0^. b) Comparison of the phase‐uncorrected FT‐EXAFS spectra from the sample before (gray) and after CA (gray intermittent) with the Cu_2_O and Cu(OH)_2_ standards (reds).

To gain further understanding about such structural changes, we evaluate the Cu K‐edge extended X‐ray absorption fine structure (EXAFS) of the system. Regardless of the exact atomic structure of the Cu sites, one can qualitatively evaluate the phase uncorrected FT‐EXAFS signal in the R‐space to distinguish the presence of Cu bonds with lighter elements (e.g., O, N, or S) between 1 and 2 Å, or with other metal atoms (e.g., Cu, Zr, Ni, or Fe) between 2 and 3 Å. Peaks located in the 2–3 Å range on the plot can be principally attributed to a Cu—Cu scattering path (Figure S13, Supporting Information). In fact, even if the presence of Zr from the MOF might contribute with Cu—Zr paths to the Cu K‐edge EXAFS, this contribution can be considered as minor. A high presence of Zr would introduce a different symmetry group that would contribute to the XANES spectrum, which was not observed. In addition, the signals from a Cu—Cu and a Cu—Zr path are expected to show below and above 2.6 Å, respectively.^[^ ^53^ ^]^ Therefore, the in‐depth analysis of the phase uncorrected FT‐EXAFS within the standard first shell Fourier analysis can help better assess their relative contributions if they coexist in the sample. The presence of disrupting Cu—Zr bonds would increase the radial distance and the Debye–Waller factor (*σ* ^2^) of the Cu—Cu scattering path significantly. Before the reaction, EXAFS of the NU1000|Cu‐tmpaCOOH principally features a peak where a Cu—O or Cu—N scattering path is expected. However, as soon as a potential is applied, we observe a peak at 2.23 Å. Fitting of the first shell of Cu—Cu yields a coordination number *N*_Cu—Cu_ of 8.35 ± 1.54 positioned at a radial distance of 2.55 ± 0.01 Å with *σ* ^2^ being 0.008 ± 0.002 (Figure S13 and Table S2, Supporting Information). These values are consistent with the hypothesis of Cu^0^ cluster formation during electrolysis. Compared to the fitting of a Cu foil (Figure S17 and Table S3, Supporting Information), the catalyst displays a relatively high crystallinity as reflected by its low *σ* ^2^ with a low *N_Cu—Cu_* error. This further points to the absence of any significant Cu—Zr contributions. According to the model of Jentys for small face‐centered cubic metallic particles,^[^ ^54^ ^]^ and assuming a homogeneous distribution of formed particles, this coordination number would correspond to 1.8 ± 1.2 nm diameter clusters (Figure S15, Supporting Information). As suggested by XANES, it also appears that the formation of these Cu^0^ clusters is more pronounced at more negative applied potentials with a corresponding decrease of the Cu—O/N peak and simultaneous increase of the Cu—Cu peak (**Figure** **3**a). The peak observed at 1.5 Å before the reaction is recovered post‐electrolysis. Qualitatively, the peak distribution of the sample EXAFS differs from the EXAFS of both Cu_2_O and Cu(OH)_2_ standards. This is consistent with the poor LCF using these references as fitting standards. In addition, the Cu species in NU1000|Cu‐tmpaCOOH before and after electrocatalysis exhibit a slight shift of the first scattering peak to smaller radial distances (Figure 3b). This could suggest either a change in the distribution of oxidation states or a contraction of the original Cu structure upon exposure to electrocatalytic conditions. Such an observation remains consistent with the hypothesis of cluster formation. Formed under reductive conditions, unprotected Cu^0^ clusters are prone to oxidation once returned to more oxidative conditions. In their work on Cu single‐atom catalysts on N‐doped carbon, Fontecave and coworkers proposed that the presence of Cu^2+^ before and after electrolysis and of Cu^0^ observed using in situ XANES was a result of the reversible formation of Cu^0^ clusters.^[^ ^49^ ^]^ However, no electrochemical characterization was presented to confirm that the Cu species before and after electrolysis are identical. Here, we can confidently claim that the Cu species after electrolysis differ from the original species, as illustrated by the vastly different redox couples identified electrochemically. This surface‐sensitive characterization method identifies two different active surface species, which correspond, for a bulk‐sensitive technique like EXAFS, to moderately different signals. However, careful MCR analysis of the XAS signal throughout the life cycle of the catalyst helps corroborate the differences identified electrochemically, with a post‐electrolysis complete disappearance of the third component representative of the Cu molecular catalyst's initial state.

**Figure 4:**
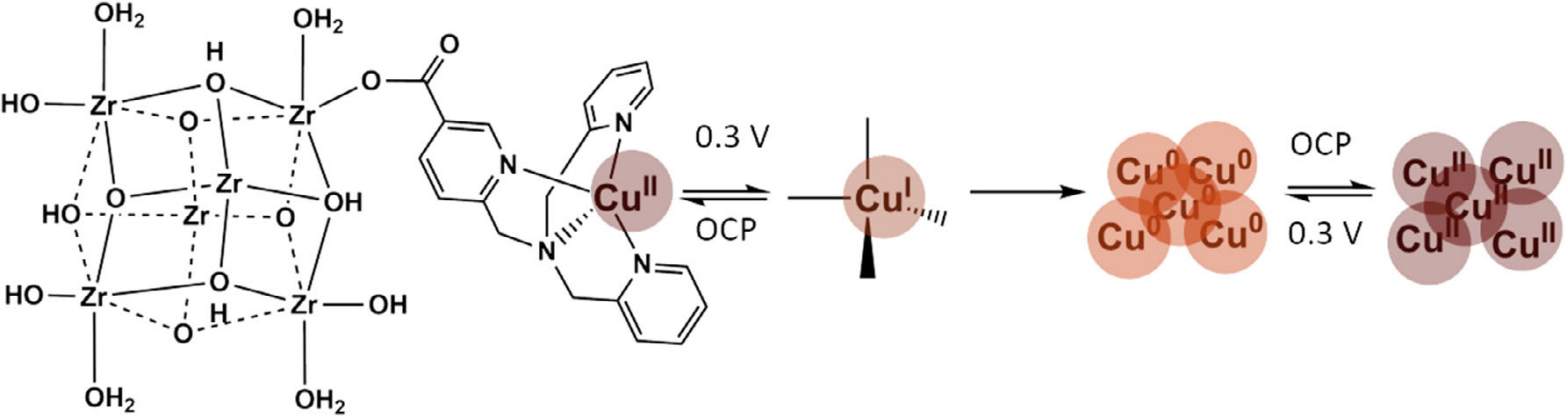
Schematic representation of the Cu species hypothesized to be formed before, during, and after ORR by NU1000|Cu‐tmpaCOOH.

Overall, our XANES and EXAFS results are consistent with the formation of Cu^0^ clusters during CA at 0.3 and −0.1 V vs. RHE. Our hypotheses extracted from these measurements are summarized in the schematic presented in **Figure** **4**. Before the experiment, the sample exhibits a Cu^2+^ character with no observable Cu—Cu distances, consistent with the presence of the Cu‐tmpaCOOH catalyst. Stable at OCP, the species present in the system progressively transform into Cu^0^ clusters when a potential of ≤0.3 V vs. RHE is applied (Figure 4). It is likely that the Cu‐tmpaCOOH catalyst first reduces to its Cu^+^ state and further reduces to Cu^0^ clusters. This transition can be explained by a partial loss of the molecular catalyst's ligands during the electrochemical reduction, as nonligated Cu^+^ species are known to easily disproportionate to Cu^0^.^[^ ^55^ ^]^ XANES and EXAFS spectra of NU1000|Cu‐tmpaCOOH measured every 10 min for the first 40 min of CA at 0.3 V vs. RHE under O_2_ atmosphere (Figure S15, Supporting Information) show that the Cu clusters are formed over time at this potential. XANES and EXAFS spectra of NU1000|Cu‐tmpaCOOH measured during CA at –0.1 V vs. RHE under He atmosphere (Figure S16, Supporting Information) show that the formation of Cu^0^ clusters is not dependent on the presence of O_2_. Therefore, we assume the formation of Cu^0^ clusters is only caused by the application of a reductive potential. When returned to OCP, these clusters readily oxidize to Cu^2+^. The Cu^2+^ state of these now oxidized clusters differs from the initial Cu^2+^ state of the Cu(II)‐tmpaCOOH molecular catalyst identified by CV and MCR analysis. According to the latter, only one out of the two Cu^2+^ components observed before electrolysis remains after the reaction. We propose that these Cu^2+^ clusters share a coordination akin to Cu(OH)_2_ (Figure S12a, Supporting Information) and are responsible for the new redox couple observed electrochemically after catalysis. Given the good reusability of the NU1000|Cu‐tmpaCOOH system,^[^ ^51^ ^]^ we expect the oxidation of Cu^0^ to these Cu^2+^ clusters to be reversible.

There is an evident difference in behavior between Cu‐tmpa in a homogeneous solution and when immobilized in an MOF. In a homogeneous solution, there is very little deposition of Cu^0^ on the electrode, and the activity is driven by the Cu‐tmpa catalyst in the +1 oxidation state.^[^ ^47^, ^48^ ^]^ The distinct selectivity of the homogeneous Cu‐tmpa with 45% H_2_O_2_ faradaic efficiency compared to its NU1000‐embedded equivalent with only 10% further supports the hypothesis of their distinct active sites. In the NU1000|Cu‐tmpaCOOH MOF, those active sites are likely the Cu^0^ atoms supported on the surface of Cu^0^ clusters that form during ORR. A probable driver for the formation of clusters in MOFs is the high local concentration of Cu atoms that can direct the equilibrium between Cu‐tmpaCOOH catalysts and Cu clusters toward the clusters. The confinement effects achieved through embedding the catalyst in an MOF were only partly effective. They were still accompanied by an unintended aggregation of the molecular Cu catalysts, thus losing their characteristics as homogeneous catalysts. These findings illustrate the necessity of employing operando spectroscopy to monitor the evolution and thus the true active state of molecular catalysts in MOFs.

## 3 Conclusion

We studied the Cu species formed in the NU1000|Cu‐tmpaCOOH MOF before, during, and after the ORR using XAS. The catalyst, with an initial Cu^2+^ oxidation state, forms Cu^0^ clusters upon application of a potential of 0.3 V vs. RHE. The extent of cluster formation increases with a more negative potential. After electrocatalysis, the Cu sites reoxidize at OCP to a Cu^2+^ state different from the initial Cu(II)‐tmpaCOOH molecular catalyst, likely Cu^2+^ clusters similar in coordination to Cu(OH)_2_. Our work distinguishes itself from similar observations made on the instability of MOF‐integrated catalytic transition metal sites during electrocatalysis.^[^ ^56^ ^]^ Instead, it highlights the dynamic nature of Cu‐based molecular catalysts in MOFs, further emphasizing the importance of the operando investigation of species formed during electrocatalysis. Through this work, we demonstrate for the first time that even when exhibiting electrocatalytic improvements, we cannot assume that the molecular catalysts embedded in MOFs remain molecular, even if the homogeneous parent systems do. As illustrated in parallel works, this seems especially important for Cu‐based catalysts whose dynamic behavior drives their reconstruction regardless of the hosting support. Such aspects exemplify the necessity of not only applying operando spectroscopy techniques to the study of molecular catalysts embedded in MOFs but also carefully correlating those characterization techniques with electrochemical measurement to verify the coherence between the chemical nature of the bulk and the surface of the studied catalytic system. We foresee that the insights gained through such an approach will provide the understanding necessary to enable future advancements in rationalized MOF‐embedded catalyst designs catered to various electrocatalytic reactions.

## Supporting Information

The authors have cited additional references within the Supporting Information.^[^ ^57^, ^58^, ^59^, ^60^, ^61^ ^]^

## Conflict of Interest

The authors declare no conflict of interest.

## Supporting information

Supplementary Material

## Data Availability

The data that support the findings of this study are available from the corresponding author upon reasonable request.
