# Supplementary material for: Surveying the Homogeneity of a Molecular Electrocatalyst Embedded in a Metal‐Organic Framework Using Operando Characterization
Source: ChemSusChem. 2025 Oct 14;18(24):e202501380. doi: 10.1002/cssc.202501380 (PMC12703425; doi:10.1002/cssc.202501380)
Supplement: Supplementary file 1 — Supplementary Material [file CSSC-18-e202501380-s001.pdf]

Supporting Information

1. **Electrochemistry**

1.1 Sample preparation and electrochemistry

Sample preparation, electrode polishing and glassware cleaning were performed as described in previous work.[1] A mixture of 2 mg NU1000|Cu-tmpaCOOH, 1 mg carbon black, 20 µL Nafion® perfluorinated resin solution in propanol and 180 µL HPLC-grade acetone was sonicated and vortexed before a 15 µL sample was drop casted onto the GC electrode. A one compartment electrochemical cell with a three-electrode setup was used. A glassy carbon rod was used as the working electrode, a gold wire as the counter electrode, a platinum mesh with H_2 bubbling as the reference electrode and a platinum wire as a condensator for electrochemical measurements without XAS (Figure 1b and Figure S1). Solutions were saturated with 1.2 mM O_2 by bubbling 15 minutes with oxygen gas, after which an oxygen flow over the solution was maintained during measurements. Currents (A) were converted to current densities (A cm^{-2}) by dividing the measured current by the electrode geometric surface area.

1.2 Sample preparation and electrochemistry for operando spectroscopy

An electrochemical cell with Al window and a volume of 5 mL was used for operando measurements (Figure S2). H23I2 carbon paper with gas diffusion layer from FuelCellStore cut into circles with a diameter of approximately 3 cm was used as the working electrode, a Pt wire as the counter electrode and a leakless miniature Ag/AgCl electrode (ET072-1) was used as the reference electrode. The MOF ink was drop casted onto the carbon paper working electrode and allowed to dry for 1 hour before use. O_2 or He saturated 0.1 M phosphate buffer pH 7 was flowed through the cell at a flow rate of 1.4 mL/min.

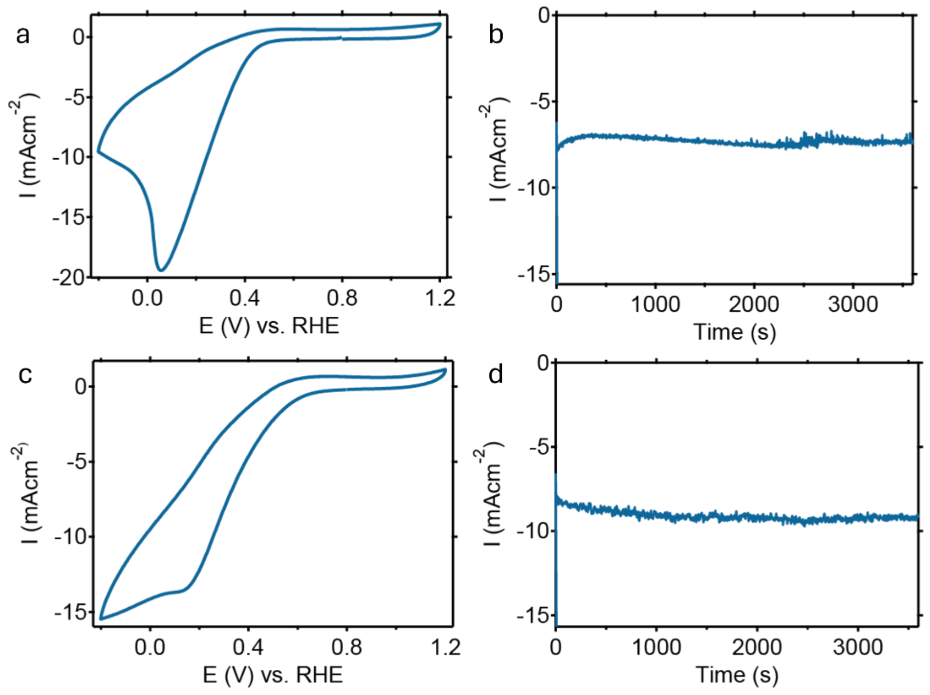

Figure S1. Electrochemistry of NU1000|Cu-tmpaCOOH in phosphate buffer pH 7. (**a**) CV at 1.12 mM O_2, (**b**) CA at 0.3 V vs. RHE at 1.12 mM O_2, (**c**) CV at 7.5 mM H_2O_2, and (**d**) CA at 0.3 V vs. RHE at 7.5 mM H_2O_2.

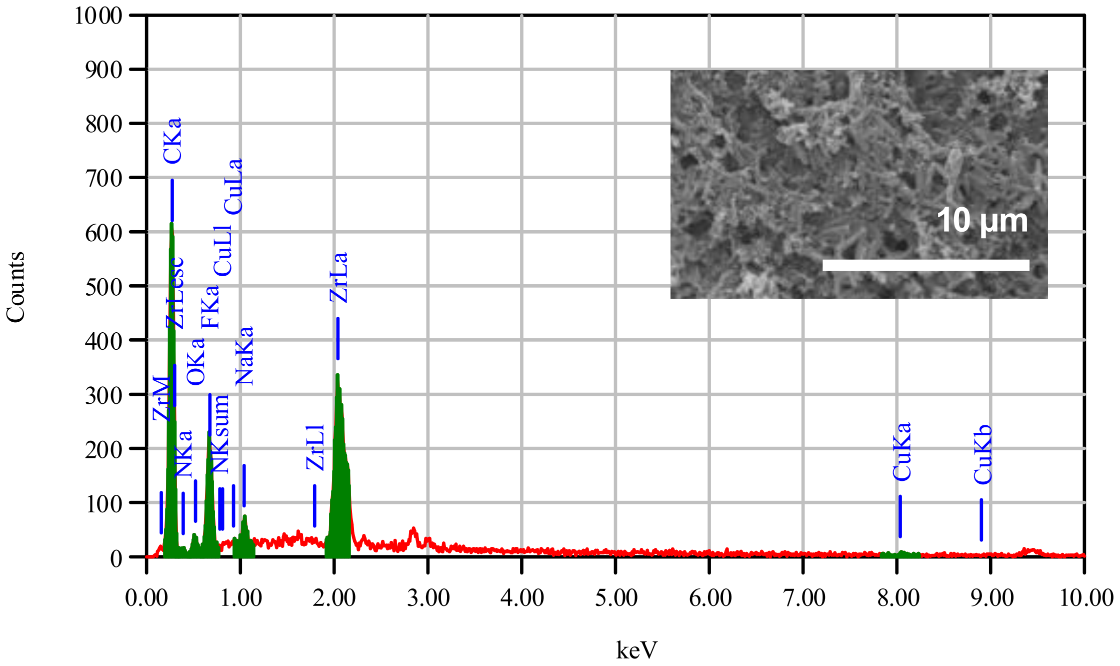

Figure S2. Scanning electron microscopy (SEM) image and the corresponding energy dispersive X-ray spectroscopy (EDX) plot of the sample after ORR using 15 kV. While Zr-specific peaks are distinguishable, the signal to noise ratio for the Cu-specific peaks is too low to be reliably quantified and mapped out.

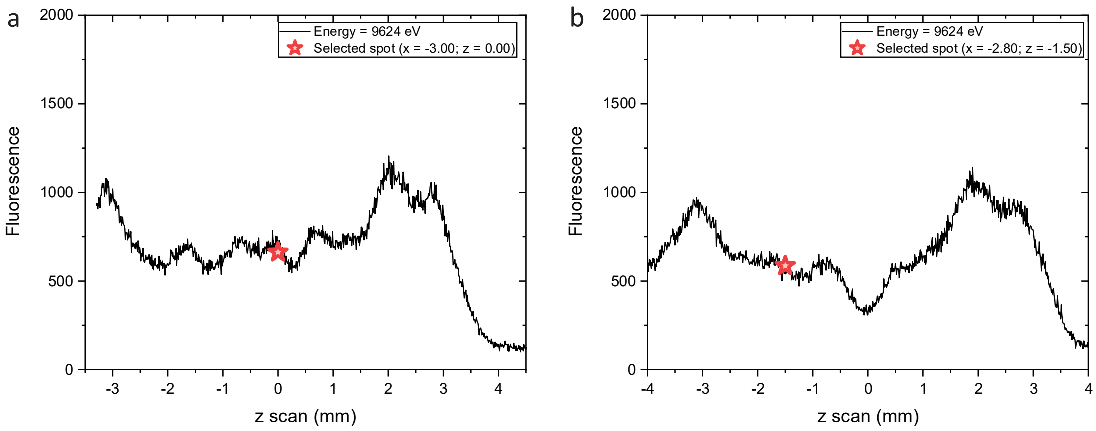

Figure S3. Fluorescence intensity measured at 9420 eV while scanning the sample on the z-axis at 2 different x-y positions. The relative fluctuations in intensity suggest a homogeneous dispersion of the Cu sites over the electrode-surface.

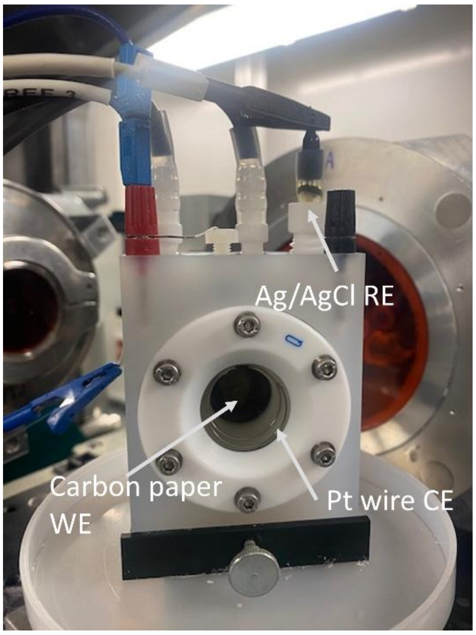

Figure S4. Electrochemical cell used for operando X-ray absorption measurements.

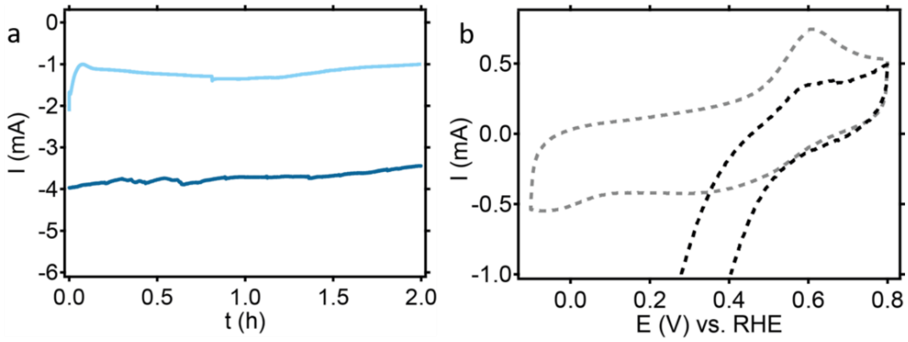

Figure S5. (**a**) CA of NU1000|Cu-tmpaCOOH under O_2 atmosphere at 0.3 V vs. RHE (light blue) and -0.1 V vs. RHE (dark blue) measured during operando XANES and EXAFS shown in Figure 2 and Figure 3. (**b**) CV under He atmosphere after CA under O_2 atmosphere (black intermittent) and after CA under He atmosphere (grey intermittent).

2. X-ray absorption spectroscopy

X-ray absorption spectroscopy (XAS) experiments were conducted at the ALBA synchrotron at beamline BL 22 CLAESS. All ex situ samples were characterized in transmission mode while all of the operando measurements were carried out in fluorescence mode using RaySpec silicon drift detector equipped with 6 Be windows. The XANES and EXAFS data processing, including energy calibration and edge step normalization, was done in Athena. The linear combination fitting (LCF) was also carried out in the same software. EXAFS fitting was performed in Artemis according to previous procedures.[2] Both programs are part of Demeter package 0.9.26.[3] The FEFF model of Cu^0 was generated for the EXAFS fitting. Only the first single scattering path to the first shell from Cu^0 was used for the fit. All FT-EXAFS presented in this work is phase uncorrected. This explains why the scattering peaks presented in R-space appear at radial distances slightly below their theoretically expected values.

The evolution of Cu phases measured with XANES was also evaluated by applying the principal component analysis (PCA) approach implemented in PyFitit code.[4] The *operando* spectra were calibrated, normalized, and then treated as a single dataset. The minimum number of pure components was set to 3 using Scree Plot approach (Fig. S9). A minimum number of pure components was used in the simplisma procedure to obtain an initial estimation for the spectra of the pure components directly from the data set.[5] Then MCR-ALS algorithm was applied with non-negativity for spectra and concentration profiles,[6] and the sum of MCR concentrations was constrained to 1.

For the operando measurements, a commercial Zahner PECC-2 3-electrodes flow cell was used. The front window (facing the X-ray and the detector) was made of Al, and the back window was made of Teflon. An Al contact cover was pressed against the carbon paper working electrode to establish the working electrode connection. The counter electrode was a Pt wire coiled against the back window of the cell. A leakless miniature Ag/AgCl (ET072-1) was used as the reference electrode. The electrolyte was flown through the electrode using a peristaltic D-25Vplus pump (Dinko Instruments). The used electrolyte was purged and saturated with either 21% O_2 + He or He only, before being introduced into the cell. All electrodes were connected to a VSP potentiostat (Biologic), and the electrochemical programs were controlled using EC-Lab.

The electrochemical cell was bolted to a stage, which was controlled remotely. Before each XAS measurement, the cell position was aligned to (1) ensure the measured spot was within a homogeneous sample distribution position and (2) optimize the XAS signal intensity. The beam size was optimized to ensure that enough Cu signal was detected while minimizing beam exposure on the sample. Overall, the beam spot size used was set to 0.5 x 0.8 mm (V x H), and the photon flux was about 10^{13} ph/s. When not measuring, the beam was turned off to minimize the risk of beam damage.

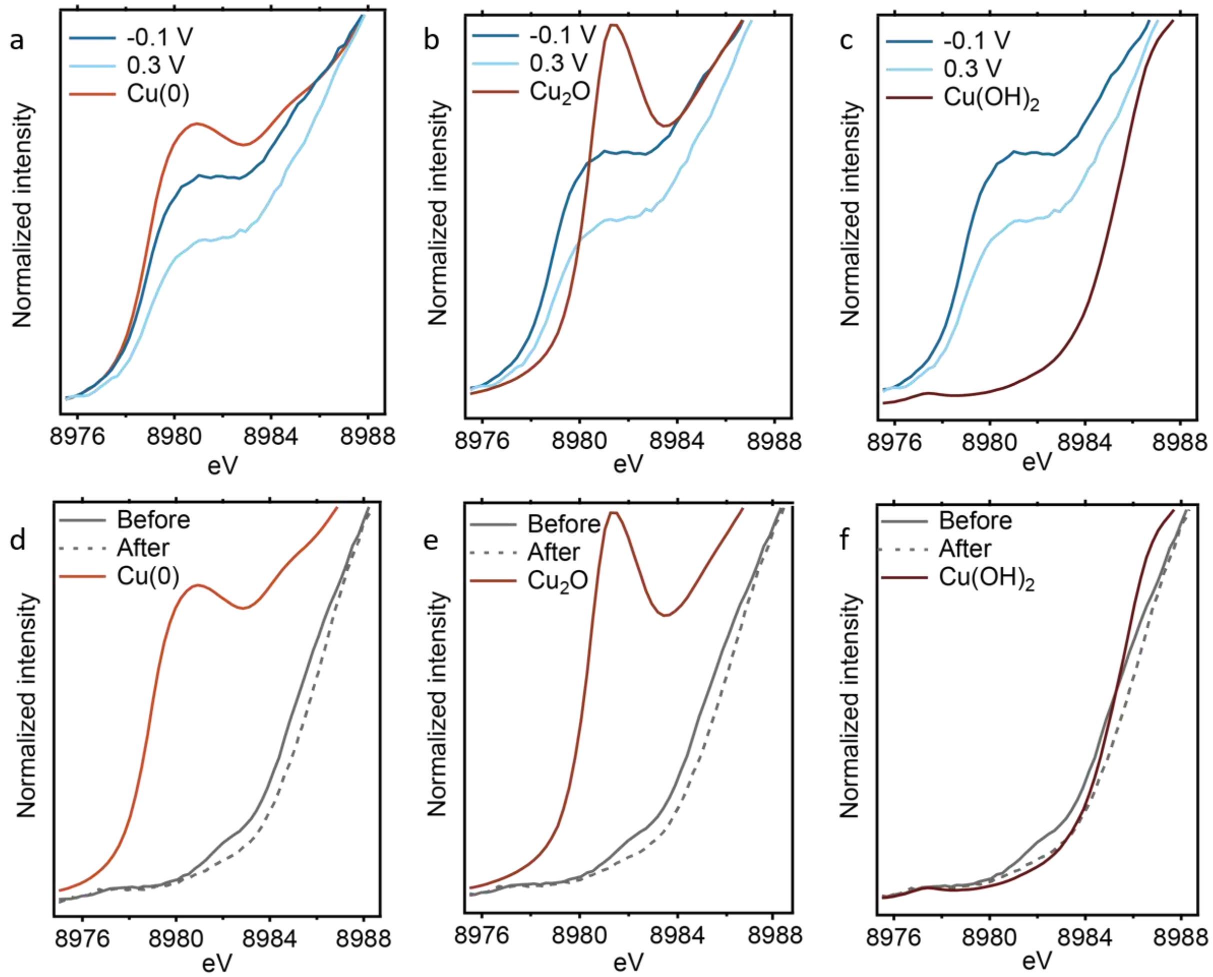

Figure S6. Comparison of the NU1000|Cu-tmpaCOOH with Cu standards in the pre-edge region of Cu K-edge XANES. (**a-c**) Under O_2 atmosphere at 0.3 V vs. RHE (light blue) and at -0.1 V vs. RHE (dark blue). (**d-f**) Before (grey) and after (grey intermittent) CA. Spectra are compared to Cu^0 foil, Cu_2O, and $Cu(OH)_2$ standards.

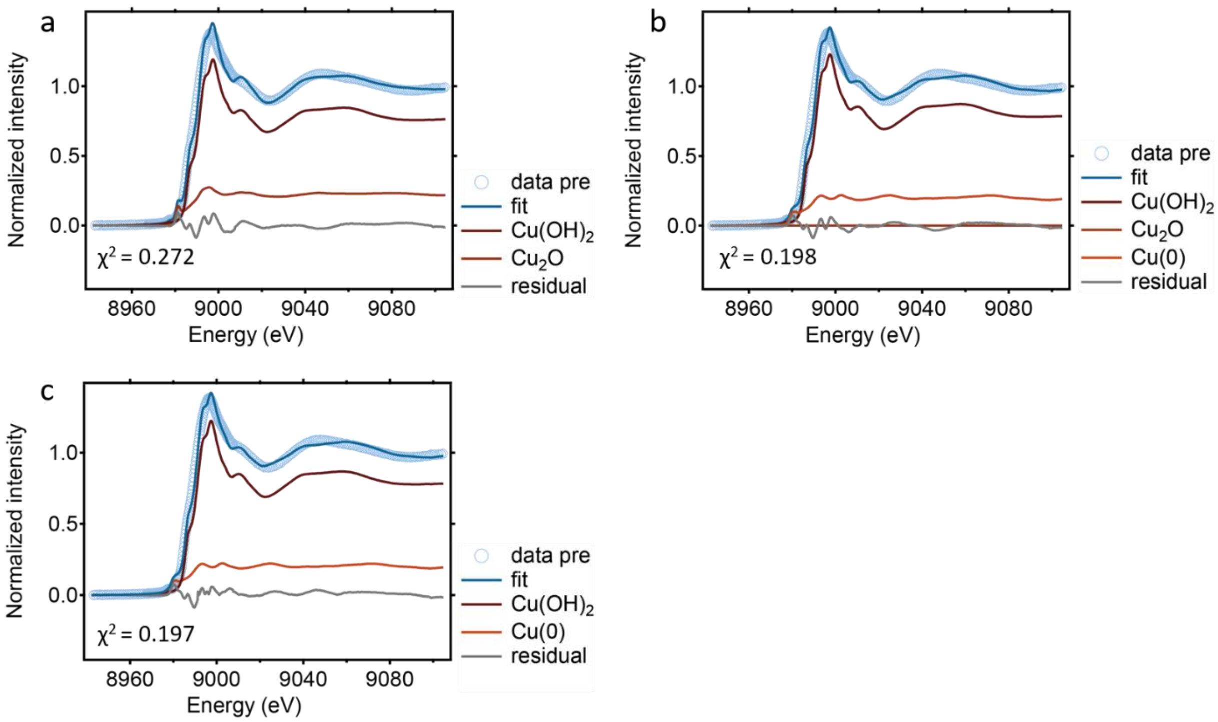

Figure S7. Linear combination fitting (LCF) of the Cu K-edge XANES spectrum of NU1000|Cu-tmpaCOOH at OCP with (**a**) $Cu(OH)_2$ and Cu_2O, (**b**) with $Cu(OH)_2$, Cu_2O, and Cu^0, and (**c**) with $Cu(OH)_2$ and Cu^0.

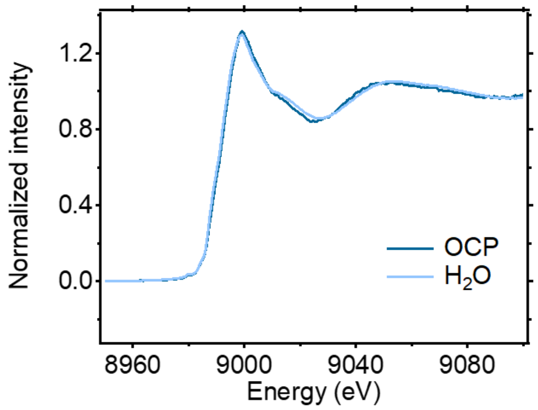

Figure S8. Cu K-edge XANES spectra of NU1000|Cu-tmpaCOOH in H_2O and in O_2 saturated phosphate buffer pH 7 at OCP.

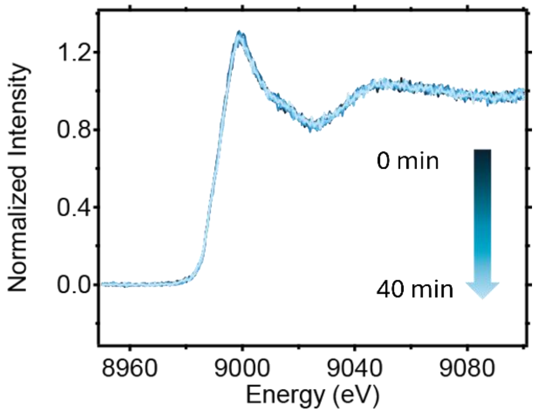

Figure S9. Cu K-edge XANES spectra of NU1000|Cu-tmpaCOOH in O_2 saturated phosphate buffer pH 7 at OCP subsequently measured, 9 scans, 5-minute long each for a total of 40 minutes.

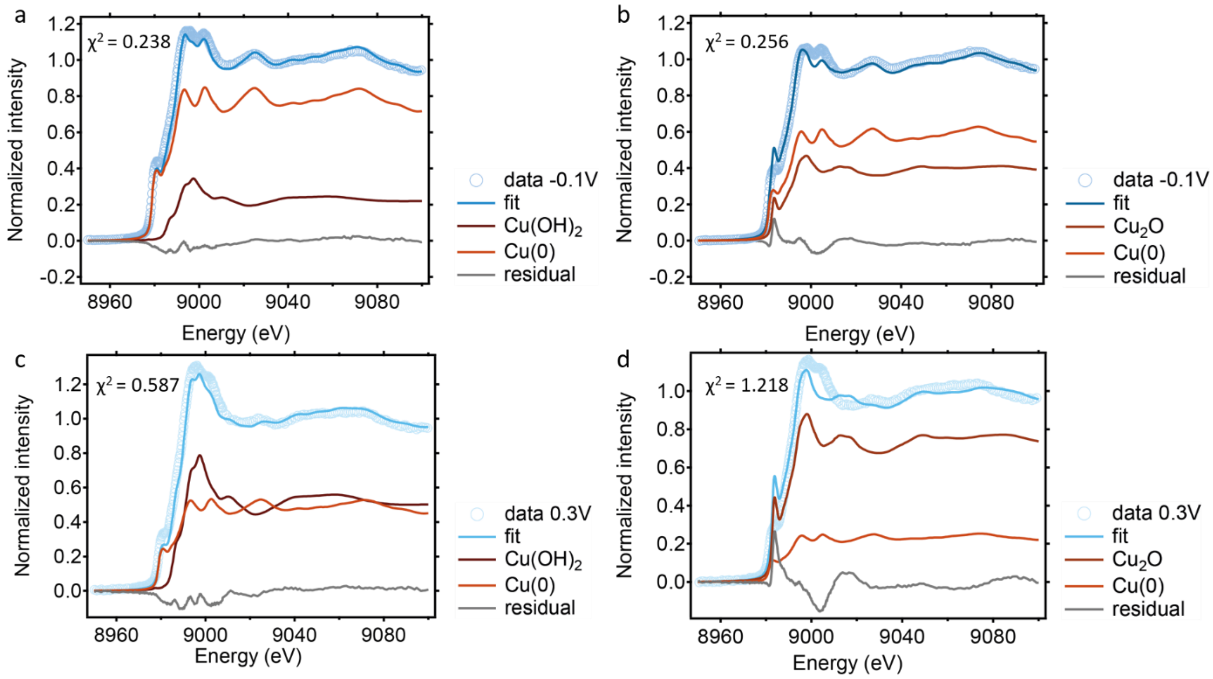

Figure S10. Linear combination fitting (LCF) of the Cu K-edge XANES spectrum of NU1000|Cu-tmpaCOOH in O_2 saturated phosphate buffer pH 7 at −0.1 V vs. RHE with (**a**) $Cu(OH)_2$ and Cu^0 and (**b**) Cu_2O and Cu^0. At 0.3 V vs. RHE with (**c**) $Cu(OH)_2$ and Cu^0 and (**d**) Cu_2O and Cu^0. The resulting fractions of Cu oxidation states are reported in Table S1.

Table S1. Fractions of Cu oxidation states found at -0.1 V and 0.3 V vs. RHE as determined from LCF in Figure S8.

**NU1000|Cutmpa-COOH O_2**	**Cu(0)**	**$Cu(I)_2O$**	**$Cu(II)(OH)_2$**	**R-factor**	**χ^2**
**-0.1 V (vs. RHE)**	0.776		0.224	0.006	0.238
	0.60	0.40		0.010	0.256
**0.3 V (vs. RHE)**	0.487		0.513	0.013	0.587
	0.24	0.76		0.039	1.218

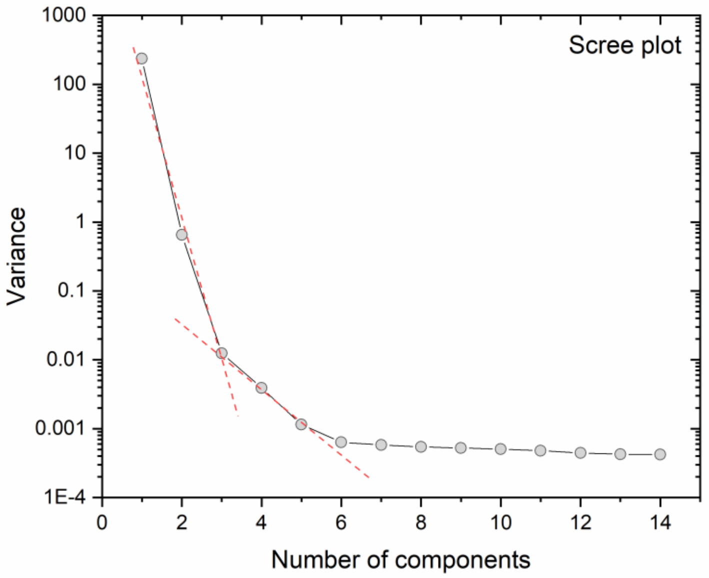

Figure S11. Variance of the Cu K-edge operando data set as a function of the number of components. The obtained MCR components were compared with standards of known oxidation state (Fig. S10). The 1st MCR component overlaps with $Cu(OH)_2$, representing Cu^{2+}. The 2nd MCR component represents metallic Cu^0 nanoparticles. The 3rd MCR component has similar features to the CuO spectrum, but does not fully overlap.

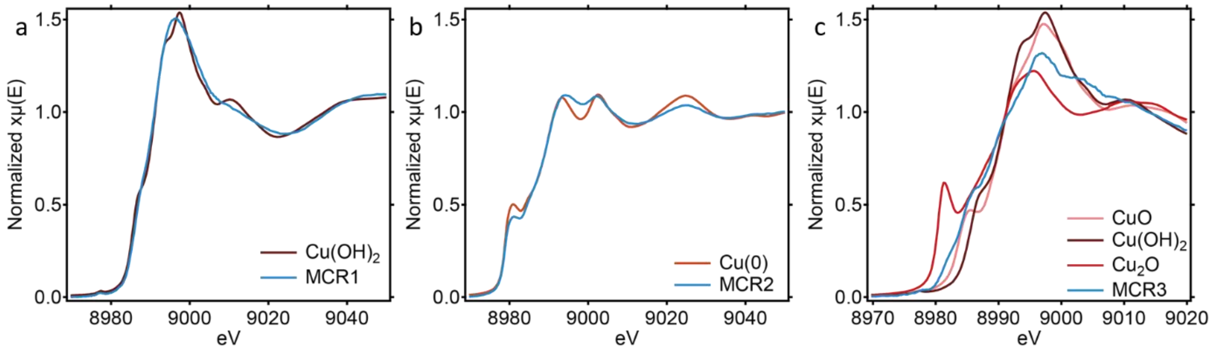

Figure S12. Comparison of the Cu K-edge XANES of the different MCR fitting elements identified for the NU1000|Cu-tmpaCOOH in O_2 saturated phosphate buffer pH 7 at −0.1 V vs. RHE with Cu standards. (**a**) MCR1 compared to $Cu(OH)_2$, (**b**) MCR2 to Cu^0 foil, and (**c**) MCR3 to CuO, $Cu(OH)_2$, and Cu_2O.

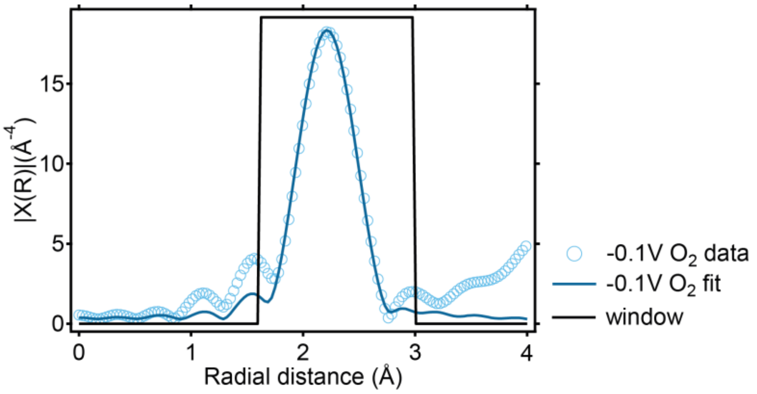

Figure S13. First shell fit of the phase uncorrected Cu K-edge FT-EXAFS of NU1000|CutmpaCOOH in O_2 saturated phosphate buffer pH 7 at −0.1 V vs. RHE. The data was fitted for 3<k<11.6 and 1.6<R<3, using solely the first Cu-Cu scattering path calculated by the FEFF model of fcc Cu^0. The negligible amount of oxide phases highlighted by the MCR analysis indicates it is unnecessary to include them in the model used for data fitting. The EXAFS-derived parameters are summarized in Table S2.

Table S2. EXAFS fitting parameters for the fit displayed in Fig. S11.

	N	R (Å)	σ^2
**Cu-Cu**	8.35±1.54	2.55±0.01	0.008±0.002
**ΔE_{Cu}**	-2.54±1.87		
**Reduced χ^2**	91.88		
**R-factor**	0.015		

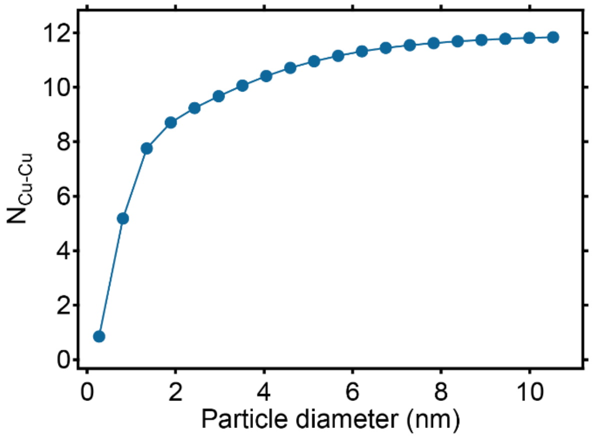

Figure S14. Coordination number N_{Cu-Cu} dependence on the particle diameter as described by Jentys for a cuboctahedral shape.[7] N_{Cu-Cu} of 8.35 corresponds to a particle diameter of ca. 1.8 nm.

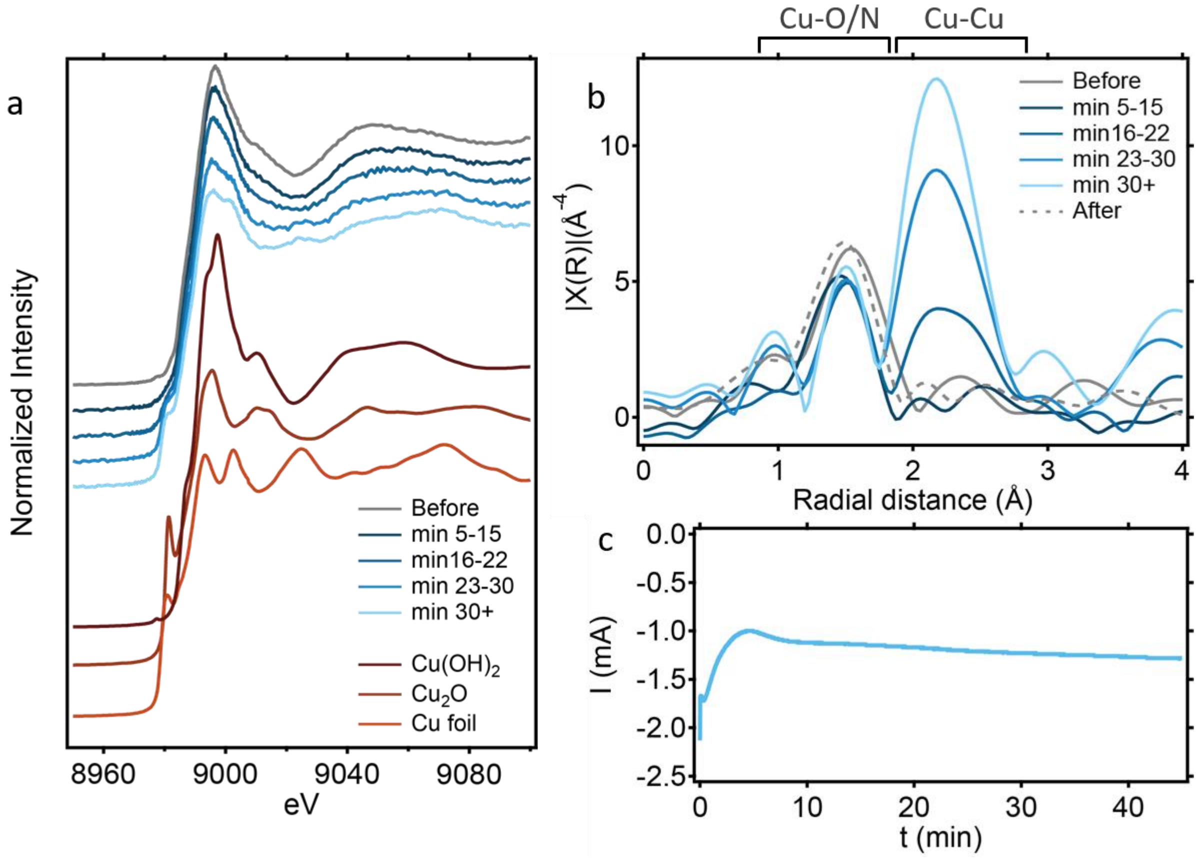

Figure S15. Cu K-edge XAS of NU1000|Cu-tmpaCOOH measured during CA at 0.3 V vs RHE under O_2 atmosphere. (**a**) XANES and (**b**) FT-EXAFS spectra captured every 10 minutes for the first 40 minutes of (**c**) CA. The XANES spectra for Cu^0, Cu^+, and Cu^{2+} references are shown in red. Each scan takes 10 minutes and is measured directly after the previous one.

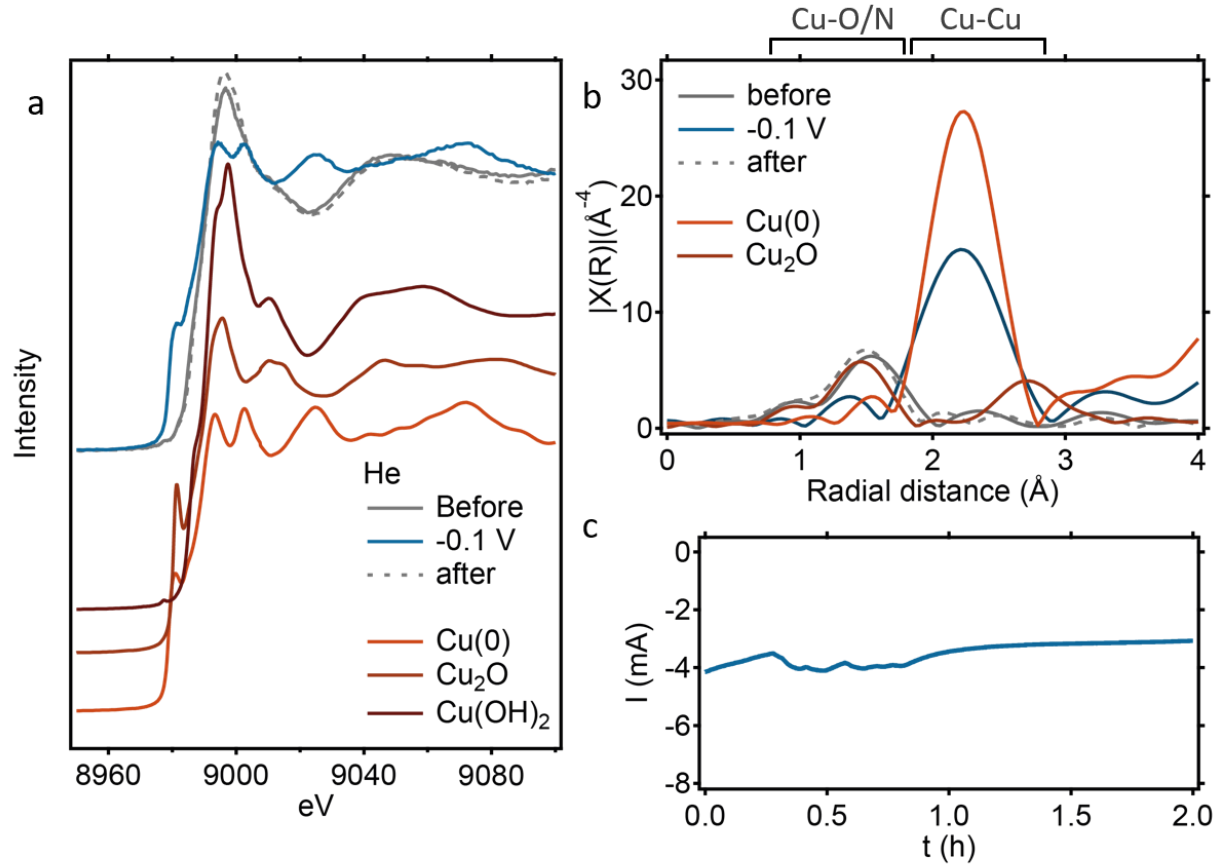

Figure S16. Cu K-edge XAS of NU1000|Cu-tmpaCOOH at −0.1 V under He atmosphere. (**a**) XANES and (**b**) EXAFS spectra measured before, during, and after (**c**) CA.

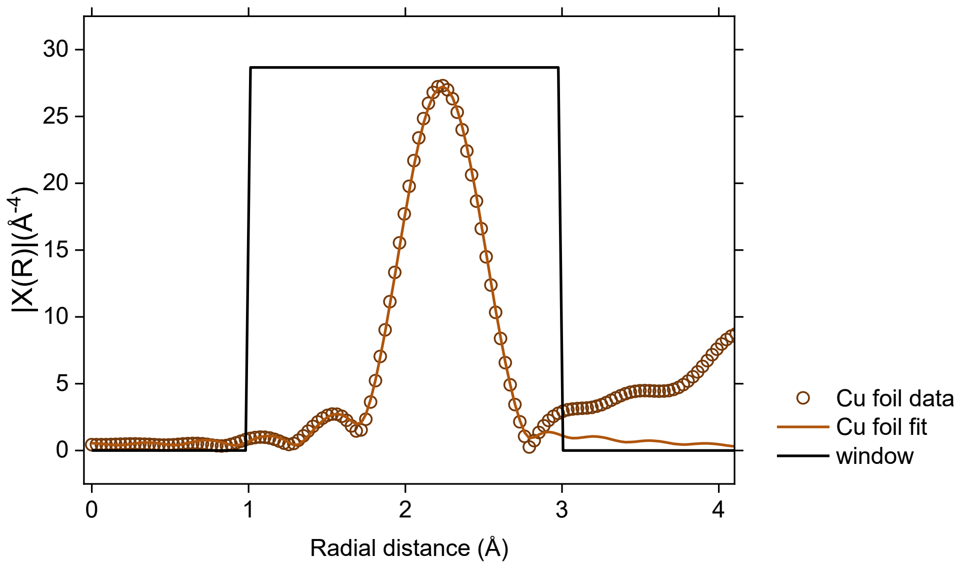

Figure S17. First shell fit of the phase uncorrected Cu K-edge FT-EXAFS of Cu foil. The data was fitted for 3<k<11.6 and 1.6<R<3, using solely the first Cu-Cu scattering path calculated by the FEFF model of fcc Cu^0. The EXAFS-derived parameters are summarized in Table S2.

Table S3. EXAFS fitting parameters for the fit displayed in Fig. S17.

	**N**	**R (Å)**	**σ^2**
**Cu-Cu**	12	2.54±0.01	0.009±0.001
**ΔE_{Cu}**	4.46±0.60		
**Reduced χ^2**	789.18		
**R-factor**	0.006		

3. References

[1] M. E. Hoefnagel, D. Rademaker, D. G. H. Hetterscheid, *ChemSusChem* **2023**, *16*, e202300392.

[2] D. Kim, N. Becknell, Y. Yu, P. Yang, *Nano Lett* **2017**, *17*, 2732–2737.

[3] B. Ravel, M. Newville, *J. Synchrotron Rad.* **2005**, *12*, 537–541.

[4] A. Martini, S. A. Guda, A. A. Guda, *Comput Phys Commun* **2020**, *250*, 107064.

[5] W. Windig, J. Guilment, *Anal Chem* **1991**, *63*, 1425–1432.

[6] A. de Juan, R. Tauler, in *Data Handling in Science and Technology* (Ed.: C. Ruckebusch), Elsevier, **2016**, pp. 5–51.

[7] A. Jentys, *Physical Chemistry Chemical Physics* **1999**, *1*, 4059–4063.
